# Improvement in the Cynaropicrin, Caffeoylquinic Acid and Flavonoid Content of Globe Artichokes with Gibberellic Acid Treatment

**DOI:** 10.3390/plants11141845

**Published:** 2022-07-14

**Authors:** Sara Lombardo, Aurelio Scavo, Gaetano Pandino, Marco Cantone, Giovanni Mauromicale

**Affiliations:** Department of Agriculture, Food and Environment (Di3A), University of Catania, 95123 Catania, Italy; sara.lombardo@unict.it (S.L.); aurelio.scavo@unict.it (A.S.); marco94caa@gmail.com (M.C.); g.mauromicale@unict.it (G.M.)

**Keywords:** *Cynara cardunculus* var. *scolymus*, gibberellic acid, caffeoylquinic acids, apigenin derivatives, luteolin derivatives, cynaropicrin, yield

## Abstract

Increasing interest has been shown in globe artichoke for pharmaceutical and food applications as a significant source of polyphenol compounds. With the aim to improve the polyphenol profile of globe artichoke, an open-field experiment is set up to study the effect of gibberellic acid (GA_3_) application on the cynaropicrin, caffeoylquinic acid and flavone levels of three genotypes (‘Apollo’, ‘Tema 2000’ and ‘Violet de Provence’), along with crop earliness, yield and bio-morphological plant response. The results indicate that GA_3_ treatment is more effective in terms of total polyphenol and caffeoylquinic acid accumulation in ‘Apollo’, regardless of the plant parts analyzed. In particular, the leaves of ‘Apollo’ were the richest source of luteolin derivatives of 5-*O*- and 1,5-di-*O*-caffeoylquinic acids. On average across the genotypes, GA_3_ treatment favored the accumulation of apigenin derivatives in the floral stem, and caffeoylquinic acid and cynaropicrin in the leaves. From the perspective of yield, GA_3_ treatment allowed us to anticipate the first harvest in each genotype, while either maintaining (‘Apollo’, ‘Violet de Provence’) or enhancing (‘Tema 2000’) the fresh weight yield. In conclusion, this study could be interesting for promoting GA_3_ usage to ameliorate the globe artichoke nutraceutical profile and to anticipate the first harvest for fresh marketing without significant yield losses.

## 1. Introduction

The globe artichoke (*Cynara cardunculus* L. var. *scolymus* (L.) Fiori = *C. scolymus* L.) is a perennial plant of the Asteraceae (ex Compositae) family, grown for its immature inflorescence (called the capitulum or head) which consists of a receptacle surrounded by thickened bracts [1]. Although Mediterranean countries such as Italy (38 Kha), Spain (16 Kha) and Egypt (14 Kha) remain the leading producers, globe artichoke is currently also grown in the Americas and Asia [2] and is globally appreciated as a culinary delicacy. The globe artichoke plant, depending on the genotype, may produce from 3–4 to 20 heads, with a mean weight ranging between 100 and 300 g depending on the genotype and its position in the plant [3]. Genotype variability has been recorded for other traits, such as plant size, leaf shape, earliness, head number per stem, shape and color of the heads, ratio of the edible parts, shelf-life, and presence of thorns or spines [4,5]. An increase in biodiversity represents the best tool to satisfy production chains (the fresh consumption and processing industries), also providing a temporal continuity to product quality, and improving farmers’ incomes [6].

The nutritional value of globe artichoke is related to its low-fat content and high amount of minerals, fiber, vitamins, and bioactive compounds [7,8,9]. In particular, globe artichoke heads accumulate more polyphenols than other vegetables [10]. The main classes of polyphenol compounds in globe artichoke plants are mono- and di-caffeoylquinic acids (including chlorogenic acid and cynarin), flavones such as apigenin and luteolin derivatives, and cynaropicrin, the main compound of sesquiterpene lactones [11]. In particular, the recovery of these bioactive compounds from large quantities of globe artichoke agro-industrial discards (nearly 80% of the total biomass and mostly represented by leaves and floral stems) could represent a valuable way to enhance sustainability and productivity, favoring a circular economy [12]. Due to globe artichoke’s reputation as a functional food and a delicacy ingredient in many recipes, it is imperative to find methods allowing the improvement of crop yield and quality to meet consumers’ demand. In this context, gibberellic acid (GA_3_) is one of the most recommended plant growth regulators widely used in the cultivation of globe artichoke to anticipate and modulate head production and increase crop yield potential. Indeed, the exogenous supply of GA_3_, through the stimulation of both cell elongation and division, allows us to accelerate and synchronize flowering and to increase yield [13,14,15]. However, some aspects, including genotype, sowing/planting time, climatic and soil conditions, the concentration of the active compound, and the frequency of application, may influence the effectiveness of GA_3_ treatments [13,15,16,17]. In addition, literature data about GA_3_ effects on globe artichoke’s performance are variable [15,18]. For example, Foury [19] highlighted that an over-supply of GA_3_ can improve the production of secondary heads, leading to a reduction in the crop’s commercial value. However, such secondary heads, being smaller than the primary ones, are widely processed as canned, frozen and ready-to-eat products. On the other side, Basnizki [20] noted a negative effect of GA_3_ treatment on the quality at harvest, given that the head’s accelerated growth results in a decreased head fresh weight, an increased length of bracts and, in some cases, a deformation in head shape. In addition, heads collected from GA_3_-treated plants are generally waterier than those from untreated ones, tend to be more liable to damage due to low temperatures or pathogen attack, and lose pigmentation [21]. To avoid such qualitative depreciation, it is crucial to adjust the rate and timing of nitrogenous fertilization [22]. In this framework, the basic assumption is that GA_3_ application, by promoting plant growth, may stress plants, inducing secondary metabolite biosynthesis. Indeed, after the treatment with GA_3_, the plants appear chlorotic and change their morphological structure. In addition, to our knowledge, the influence of GA_3_ application on the level of polyphenols has not been studied extensively and systematically yet for the globe artichoke. Furthermore, due to the high content of polyphenols from agro-industrial by-products (e.g., leaves and floral stems), it is necessary to study how GA_3_ treatment may impact on the accumulation of these bioactive compounds in different parts of the head (leaf, floral stem and receptacle). Considering all these aspects, the aim of this research is to assess the efficiency of GA_3_ application on the polyphenol profile of three globe artichoke genotypes collected at two contrasting harvest times (mid-winter and early spring), along with the effectiveness of GA_3_ in terms of earliness and crop yield traits.

## 2. Results

### 2.1. Polyphenol Profile Characterization

The polyphenol profiles of each globe artichoke plant’s parts were significantly influenced by the main factors studied (Table 1). For all parameters studied, the genotype and the harvest time significantly contributed to the total variance. With the exception of the content of Tot Api in the receptacle and Tot CQA in the leaf, the GA_3_ treatment significantly impacted the polyphenol profiles. In particular, it represented the main factor for Tot Phe in the floral stem and for both Tot Api and Tot Lut in the leaves (Table 1). The interaction of GA_3_ treatment with either genotype or harvest time was significant in 8 and 9 out of 13 cases, respectively.

For the floral stem, the interaction ‘GA_3_ treatment × harvest time’ was significant for all traits, with the exception of Tot Lut (Table 1). At both harvest times, GA_3_ samples reached higher values of Tot CQA and Tot Api than the control ones (Table 2). Particularly, at H1 the floral stems from the GA_3_-treated plants had a doubled Tot CQA compared to those from the untreated plants (14.65 vs. 7.23 g kg^−1^ DM). By contrast, Tot Phe was higher for the floral stems from GA_3_-treated plants only when collected at H1. This trend was also evidenced for the content of Tot CQA and Tot Phe in the receptacle, at least for H1. Additionally, for the leaves, GA_3_ samples displayed a higher Tot Phe than the control ones at H1 as a response to the accumulation trend of Tot Lut. Conversely, the cynaropicrin content in the leaves was higher in the GA_3_ samples for both harvest times. 

According to the significant ‘GA_3_ treatment × genotype’ interaction, all the studied genotypes presented a higher Tot CQA in the floral stems from GA_3_-treated plants (Table 3). Conversely, Tot Lut levels were higher in the control samples, at least for ‘Tema 2000’ and ‘Violet de Provence’. The latter also showed any statistical differences between untreated and treated plants with GA_3_ in terms of Tot Phe. Indeed, ‘Apollo’ displayed a major increase (+61%) due to GA_3_ treatment. For the receptacle, the interaction of ‘GA_3_ treatment × genotype’ was significant only for Tot CQA and Tot Phe. In both cases, ‘Apollo’ had higher values of such parameters when treated with GA_3_, while ‘Tema 2000’ and ‘Violet de Provence’ reached higher levels in the control samples. The leaves of ‘Apollo’ and ‘Violet de Provence’ showed higher Tot Phe and Tot Lut when treated with GA_3_, while these traits in ‘Tema 2000’ were not influenced by treatments. For all the genotypes, the cynaropicrin amount in the leaves was significantly higher after supplying GA_3_ to the globe artichoke plants. 

A significant ‘genotype × harvest time’ interaction was displayed for all the compounds detected in the floral stem (Table 1). In particular, ‘Tema 2000’ and ‘Violet de Provence’ accumulated more Tot Phe and Tot CQA in mid-winter than in early spring, while the opposite was true for the Tot Api and Tot Lut levels (Table 4). All the genotypes also presented an almost doubled Tot Lut content in the receptacle in early spring compared to mid-winter. With the exception of ‘Apollo’, the genotypes studied reported a higher Tot CQA at early spring. For the leaves, ‘Violet de Provence’ and ‘Tema 2000’ highlighted higher values at mid-winter, while an opposite behavior was observed for ‘Apollo’ (Table 4). Cynaropicrin level was higher in ‘Apollo’ and ‘Tema 2000’ in early spring, whereas no differences between harvest times were reported by ‘Violet de Provence’.

Considering individual polyphenol compounds in the examined plant parts, a clear significant effect of the factors under study can be seen, i.e., genotype, treatment and harvest time (Table 5). In particular, the GA_3_ treatment significantly increased all the CQAs present in the floral stem, but especially 3,5- and 1,5-di-CQAs. Additionally, Api-7-glu and Api-7-mal levels were enhanced by GA_3_ application, while Lut-7-glu and Lut-7-glr were higher in the control samples. In the receptacles, by contrast, 3,5- and 1,5-di- CQAs were higher in the untreated samples than in the GA_3_ ones. Looking at the Api derivatives, only Api-7-glu was higher in the GA_3_ samples, whereas no differences between treatments were achieved for the Lut derivatives. GA_3_ treatment significantly increased only the foliar content of cynaropicrin, Api-7-glr and Lut-7-glr. By contrast, 5-CQA and 1,5-diCQA were higher in the control samples.

Harvest time had a statistical effect on the accumulation of polyphenol compounds and cynaropicrin (Table 5). In the receptacles, 5-CQA, 3,5- and 1,5-diCQAs as well as Api and Lut-7-glu were higher in early spring than in mid-winter. By contrast, Api-7-rut, Api-7-glu and Api-7-glr were more abundant at mid-winter. An opposite trend was observed for the floral stem, since 5-CQA, 3,5- and 1,5-diCQAs were higher at H1. By contrast, flavones such as Lut-7-glr, Lut-7-glu and Api-7-mal were higher in the sample collected in early spring. The leaves evidenced highest Api-7-glr and 5-CQA at mid-winter, while 1,5-diCQA, Api-7-glu and Lut reported major values at early spring. 

Among genotypes, ‘Violet de Provence’ was the most efficient accumulator of 5-CQA, 1,5-diCQA, Api-7-rut, Api-7-glu and Lut-7-glu in the receptacle (Table 5). ‘Apollo’ receptacles best performed only in terms of Lut-7-mal and Api. ‘Violet de Provence’ presented more 5-CQA in the floral stem than the other genotypes. By contrast, ‘Tema 2000’ floral stems were the richest source of Api-7-mal, Lut-7-mal and Lut-7-glr. In addition, it was distinguishable for having the highest levels of cynaropicrin, Api-7-glu and Api-7-glr in the leaves. However, this was interesting to underline as ‘Apollo’ displayed the highest amounts of 5-CQA, 1,5-diCQA and Lut-7-glu in the leaves.

### 2.2. Earliness, Head Characteristics and Yield Response

The ANOVA test revealed that for four out of the six bio-morphological traits under study the ‘GA_3_ treatment × genotype’ interaction explained over 23% of the total variance (Table 6). GA_3_ treatment allowed us to anticipate the harvest of the main head in all the genotypes studied (Table 7). In particular, the harvest of the first head was about 40 days earlier in the GA_3_-treated plants of ‘Apollo’, while in those of ‘Tema 2000’ and ‘Violet of Provence’ it was anticipated by about 27 and 8 days, respectively. However, the GA_3_ application increased the fresh weight yield only for ‘Tema 2000’, compared to the control treatment (1.6 vs. 1.1 kg heads plant^−1^). This appeared in line with the significant increase in the number of heads plant^−1^ collected from the GA_3_-treated plants of such genotype. By contrast, the GA_3_ treatment proved to be less efficient in improving the number of heads of plant-1 for ‘Apollo’ and ‘Violet of Provence’. 

The mean head fresh weight, deprived of the floral stem, was significantly reduced by GA_3_ treatment in ‘Tema 2000’ and ‘Violet de Provence’, while no significant differences were highlighted for ‘Apollo’ (Table 7). By contrast, the edible fraction percentage was higher in the GA_3_-treated samples of ‘Apollo’ than in the untreated ones (Table 7). No significant differences between the treatments were found for ‘Violet of Provence’ and ‘Tema 2000’. The head length/width ratio, an important index of head shape, was significantly influenced by the ‘treatment × genotype’ interaction (Table 7). In all the studied genotypes, it was significantly higher in the heads collected from the GA_3_-treated plants, as especially observed for ‘Apollo’.

## 3. Discussion

The present study highlights the impact of GA_3_ treatment on polyphenol and cynaropicrin content in three globe artichoke genotypes, along with crop earliness, yield, and head morphological traits. Indeed, the great interest in globe artichoke production as a food source of pharmaceutically bioactive compounds has led producers to find crop management practices able to contemporaneously accelerate the early production of heads, thus obtaining increased economic benefits from higher product prices and ameliorating head quality with particular emphasis on phytochemical levels. In this study, specific attention was directed to elucidate the effects of GA_3_ treatment on polyphenol accumulation in the receptacle and waste fractions, such as the leaves and floral stem. The plant growth-promoting effects induced by GA_3_ application may represent an abiotic stressor for plants and, thus, a positive impact may be predicted on the improvement of the qualitative and quantitative polyphenolic profiles, as has been established by some authors [18,23,24]. Accordingly, in the present study, the GA_3_ application was able to ameliorate the amount of tot Phe with respect to the control, although a variable response depended on either harvest time or genotype. With regard to harvest time, at mid-winter the GA_3_ treatment significantly increased the tot Phe content in each plant’s studied parts, while at early spring this was observed only for the floral stem. Furthermore, at both harvest times, GA_3_ treatment favored the accumulation of tot Api in the floral stem and cynaropicrin in the leaves, as well as the tot CQAs in the leaves collected at mid-winter. Our results partly comply with those obtained by Sharaf-Eldin et al. [18], who showed that GA_3_ increased phenolic compounds in leaves and decreased them in the receptacles of globe artichoke. This discrepancy could be explained by the significant ‘GA3 treatment × harvest time’ interaction reported here that was not considered by Sharaf-Eldin et al. [18]. Our hypothesis is corroborated by Pandino et al. [25], who observed a fluctuation in polyphenol compound accumulation across the growing season. 

Behind the harvest time, the genotype might also have had an effect on the effectiveness of GA_3_ treatment, as revealed by the significant ‘GA_3_ treatment × genotype’ interaction in 8 out of 13 cases. On the whole, we can conclude that GA_3_ treatment was more effective in improving the polyphenol profile of ‘Apollo’ than that of the other two genotypes. Particularly, ‘Apollo’ evidenced higher tot Phe levels in GA_3_-treated plants as a consequence of the enhanced tot Lut in the leaves and tot CQAs levels in both the floral stem and the receptacle. This suggests its potential for fresh consumption as a significant dietary source of both CQAs and flavonoids. In contrast, GA_3_ application lowered the level of tot Lut in the floral stems, and tot Phe and tot CQAs in the receptacles of both ‘Violet de Provence’ and ‘Tema 2000’, making them more suitable for industrial food applications. These genotypes benefited from GA_3_ treatment in a few cases, e.g., a higher amount of tot CQAs in the floral stem. Interestingly, all the studied genotypes experienced a higher cynaropicrin level when collected from GA_3_-treated plots. A different genotypic behavior may be imputed to the presence of several isozymes of quinate hydroxycinnamoyl transferase in globe artichoke genotypes [26]. 

Considering the individual compounds, the GA_3_ treatment appeared effective in improving the level of Api-7-mal in the floral stem and Api-7-glr in the leaves. The GA_3_ application also increased the cynaropicrin level in the leaves, as well as that of Api-7-glu in both the floral stem and receptacle. For the Lut derivatives, a discrepant trend was observed in the floral stem and leaves, since GA_3_ treatment was able to increase Lut-7-glu and Lut-7-glr in the leaves while decreasing them in the floral stem. This is interesting considering that leaves greatly contributed to the total amount of waste material from globe artichoke cultivation and processing. From the perspective of circular economy, the recovery of these compounds from leaves could represent an eco-sustainable way to valorize huge amounts of waste materials from globe artichoke cultivation. In addition, such findings are relevant since globe artichoke is a significant dietary source of these compounds and their conjugates, compared to other species [25]. Our results are in line with those from Giménez et al. [24], highlighting that the Lut derivatives content was improved by the GA_3_ treatment. Conversely, these authors found that GA_3_ treatment decreased the levels of all the CQAs as compared to the untreated heads, with the exception of two minor phenolic compounds (3-CQA and 1,3-di-CQA). These different responses may depend on several factors, i.e., meteorological conditions and crop management practices, including the timing of GA_3_ application, as well as maturity stage and the metabolic processes occurring during the shift from vegetative to reproductive growth.

It is, however, essential to combine the improvement of the nutraceutical profile of globe artichoke due to GA_3_ treatment with repercussions on the production level. In this framework, GA_3_ application has been shown to promote considerable earliness in globe artichoke heading, and thus harvest timing is crucial [27,28,29,30]. Many previous studies have revealed a certain variability among globe artichoke genotypes [12,31,32]. Accordingly, in our experiments, the GA_3_ treatment guaranteed the earlier production of heads, as especially observed for ‘Apollo’ and ‘Tema 2000’. Particularly, it was interesting that GA_3_ shifted part of the head harvest of ‘Apollo’ into late autumn, while untreated control plants yielded from late winter only. However, the GA_3_ treatment significantly enhanced the number of heads of plant^−1^ only in ‘Tema 2000’, which consequently displayed an increased fresh yield. On the other hand, the GA_3_ treatment ensured the same fresh total yield as the control in the other two examined genotypes due to the counterbalancing effect of GA_3_ application both in terms of the number of heads of plant^−1^ and the fresh head weight. Despite the positive effects of GA_3_ application on flower initiation and early harvest, a negative impact on head size, fresh weight and total yield was previously documented [33,34]. In our study, the GA_3_ treatment decreased the head weight with an evident effect on ‘Tema 2000’ and ‘Violet de Provence’, which were characterized by a longer productive period than ‘Apollo’. However, the production of smaller heads may be valorized as canned, frozen and ready-to-eat products. Regardless of genotype, the incidence of the receptacle to the total head fresh weight, an important index of yield potential in terms of edible fraction, ranged from 20 to 25%. A higher receptacle incidence, as displayed by GA_3_-treated samples of ‘Apollo’ and ‘Violet de Provence’, represents a desirable result for both fresh consumption and processing. In addition, only a slight deformation of head shape, expressed by the length/diameter ratio, was experienced by the GA_3_-treated heads in all the studied genotypes. According to Basnizki [20], indeed, accelerated growth due to GA_3_ application may result in an increased bract length.

## 4. Materials and Methods

### 4.1. Experimental Field Design, Meteorological Conditions, Soil Characteristics, Crop Management and Plant Material 

The field trial was conducted in the 2018–2019 growing season on a private farm located at the Ramacca (37° 26’ N, 14° 12’ E, 120 m a.s.l.) in the Catania Plain, which is a typical area for globe artichoke production in Italy. This area has a typical Mediterranean climate, characterized by mild and scarcely rainy winters and dry and windy summers. The soil type is vertic xerofluvents [35]. The soil characteristics were: clay 42%, silt 22%, sand 36%, organic matter 15 g kg^−1^, pH 8.0, total nitrogen 1.3 g kg^−1^, available P_2_O_5_ 7.0 mg kg^−1^, and exchangeable K_2_O 104 mg kg^−1^. A meteorological station (Mod. Multirecorder 2.40; ETG, Firenze, Italy) located on the experimental field was used to daily record the air temperatures and rainfall during the growing season. The mean maximum monthly temperature ranged between 12.8 °C (in January) and 25.5 °C (April) and the mean minimum between 5.9 °C (January) and 13.4 °C (May). The total rainfall during the crop cycle was 181 mm, with the highest recorded monthly rainfall (88 mm) being in February.

The experiment was arranged in a randomized split plot design with three replications including three globe artichoke genotypes (‘Tema 2000’, ‘Violet de Provence’ and ‘Apollo’) as the main plots, and two treatments (treated with GA_3_ vs. untreated control) as the sub-plots. ‘Tema 2000’ is a cultivar that has been cropped in Sicily for the last 20 years and is characterized by high earliness (harvest time from November to May), deep violet bracts, and conical-shaped heads; ‘Violet de Provence’ is an early-reflowering multiclone genotype that is actually widespread across all of the Mediterranean Basin, producing elongated green heads with purple shades; ‘Apollo’ is a ‘Romaneschi’-type genotype with spherical heads with deep violet bracts that is late-harvested by the end of January. 

Crop planting by ‘ovoli’ (semi-dormant offshoots) was carried out manually in the field on 2 August 2018, adopting a planting density of 1.0 plants m^−2^. In consideration of the different earliness and sensitivity to GA_3_ treatment of each genotype studied, a different schedule of GA_3_ application according to standard local custom was adopted. In particular, ‘Violet di Provence’ and ‘Tema 2000’ received two applications of the commercial product 86 and 108 days after the transplanting date (ATD) at a concentration of 0.01 g L^−1^, while for ‘Apollo’ GA_3_ was applied three times with increasing concentrations equal to 0.03–0.06 and 0.1 g L^−1^ at 96, 117 and 138 days ATD, respectively. Aqueous solutions of GA_3_ (Berelex^®^ 40 SG, Syngenta, Milan, Italia S.p.A.) were acidified to pH 4 by urea phosphate [36] and then applied early in the morning when plants were turgid. Each application was made using a hand-sprayer on the leaves until runoff. According to the different plant dimensions, the spray volumes were 150 and 200 mL plant^−1^ for the 1st and 2nd applications in ‘Violet di Provence’ and ‘Tema 2000’, respectively; 200–250 and 300 mL plant^−1^ were supplied at the 1st, 2nd and 3rd treatments for ‘Apollo’, respectively. 

During late spring the soil was tillaged with 35 cm deep ploughing following by harrowing. All the plots were optimally fertilized (consisting of 180, 140, and 150 kg ha^−1^ of N, P_2_O_5_, and K_2_O), following the indications from the Regione Siciliana [37] and the NPK availability of the experimental soils. Fertilization with P and K was performed before planting, and superphosphate (18% P_2_O_5_) and potassium sulphate (51% K_2_O) were incorporated into the top 30 cm soil layer. Nitrogen was applied by liquid fertilization seven times (in equal amounts every 21 days from August to December and from mid-February until April), using a liquid fertilizer Nitroplus 32 (32% N). Drip irrigation was supplied from planting to mid-October when the accumulated daily evaporation net of rain (measured from an unscreened class A pan evaporimeter near the crop) reached 40 mm (corresponding to 50% of available soil water content at 0–35 cm depth). Weed and pest control were conducted as per local custom, and lateral offshoots were removed twice, in November and February, leaving only one offshoot per plant. 

### 4.2. Earliness, Head Morphology, and Yield Responses

Heads (main and secondary) were harvested on a weekly (autumn and winter) or 4-day (spring) basis when commercially mature (stage “D”), regardless of size and with flower buds of about 2 mm [15]. According to the custom of Italian farmers, all heads were harvested with the floral stems including 2–3 attached leaves. After removing the floral stem, by cutting 0.5 cm under the receptacle, all heads were weighed to determine their fresh weight. The maximum width (W) and length (L) of each head were measured to calculate the head length/width ratio (L/W), an important index of head shape. Then, the bracts were removed from each selected head to separate the edible fraction (receptacle) in order to calculate the percentage incidence of the receptacle fresh weight on the entire head weight.

At the end of growing season, the following variables were calculated on 10 plants per treatment, genotype and replicate: earliness (as an index of commercial competitiveness of the crop, and expressed as the number of days between transplanting and the harvest of the main head), the total number of heads collected per plant, and the yield (expressed as kg fresh weight of heads per plant).

### 4.3. Polyphenol Profile Characterization

For each genotype, a subsample of at least 10 heads (including the floral stem with 2–3 leaves attached) per replicate and treatment was harvested for mid-winter and early spring, with the aim to study the influence of harvest time on the polyphenol profile. From each head, the bracts were removed to obtain the receptacle. Then, receptacles, floral stems and leaves were separately lyophilized using a Christ freeze drier (Osterode amHarz, Germany) and stored at −20 °C until analysis.

The extraction procedure for caffeoylquinic acids (CQAs) and flavones was carried out as proposed by Pandino et al. [38]. Each extract (20 μL) was then analyzed using a series 1200 HPLC (Agilent Technologies, Palo Alto, CA, USA) equipped with ChemStation software (version: B.03.01). Separations were achieved on a Zorbax Eclipse XDB-C18 (4.6 × 50 mm; 1.8 µm particle size) operated at 30 °C with a 0.2 μm stainless steel in-line filter. The mobile phase was 1% formic acid in water (solvent A) and in acetonitrile (solvent B) at a flow rate of 0.5 mL min^−1^. The gradient started with 5% B to reach 10% B at 10 min, 40% B at 40 min, 90% B at 50 min, 90% B at 58 min. 

Cynaropicrin extraction from leaves was performed according to Scavo et al. [39]. The mobile phase was 0.1% formic acid in water (solvent A) and in acetonitrile (solvent B) at a flow rate of 0.3 mL min^−1^. The gradient started with 5% B to reach 10% B at 10 min, 30% B at 25 min, and 40% at 30 min. 

**Figure 1:**
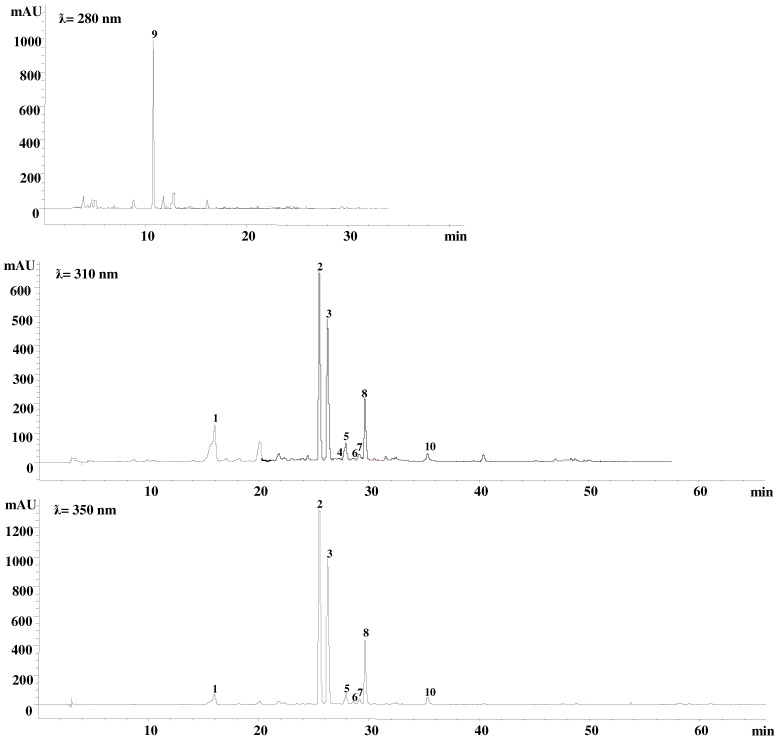
HPLC/DAD chromatograms at different wavelengths of leaf globe artichoke extracts: 5-*O*-caffeoylquinic acid (1); luteolin-7-*O*-glucoside (2); luteolin-7-*O*-glucuronide (3); 3,5-*O*-dicaffeoylquinic acid (4); 1,5-*O*-dicaffeoylquinic acid (5); apigenn-7-*O*-rutinoside (6); apigenn-7-*O*-glucoside (7); luteolin malonylglucoside (8); cynaropicrin (9); luteolin (10).

Chromatograms were recorded at 280, 310 and 350 nm from the diode array data collected between 200 and 600 nm. The identification of each compound was performed according to retention time, UV spectra, and data available in the literature [40,41]. Quantification was performed using a calibration curve with the available standards. In particular, mono- and dicaffeoylquinic acids (CQA and di-CQA, respectively) were calculated using chlorogenic acid and cynarin as references, respectively. Apigenin and luteolin conjugates were quantified as apigenin-7-*O*-glucoside and luteolin-7-*O*-glucoside, respectively. The analyses were carried out in triplicate (*n*  =  3) and the data, expressed as g kg^−1^ of dry matter (DM), are presented as mean values  ±  standard error. In particular, the following compounds were detected: 5-CQA: 5-*O*-caffeoylquinic acid (or chlorogenic acid); 3,5-diCQA: 3,5-di-*O*-caffeoylquinic acid; 1,5-diCQA: 1,5-di-*O*-caffeoylquinic acid; 4,5-diCQA: 4,5-di-*O*-caffeoylquinic acid; API-7-rut: Apingenin 7-*O*-rutinoside; API-7-glc: Apigenin 7-*O*-glucoside; API-7-glr: Apigenin 7-*O*-glucuronide; API-7-mal: Apigenin 7-*O*-malonylglucoside; API: Apigenin; LUT-7-glc: Luteolin 7-*O*-glucoside; LUT-7-glr: Luteolin 7-*O*-glucuronide; LUT-7-mal: Luteolin 7-*O*-malonylglucoside; and LUT: Luteolin. In Figure 1, the HPLC/DAD chromatograms at different wavelengths of leaf globe artichoke extracts are reported. We calculated the content of total caffeoylquinic acid (Tot CQA), total apigenin derivatives (Tot Api), and total luteolin derivatives (Tot Lut) as the sums of the individual compounds belonging to the relative classes, while total measured polyphenols (Tot Phe) were calculated as the sum of Tot CQA, Tot Api and Tot Lut.

Reagents and solvents were purchased from VWR (Leighton Buzzard, UK) and were of analytical- or HPLC-grade. Apigenin 7-*O*-glucoside, apigenin, luteolin 7-*O*-glucoside, luteolin, 5-*O*-caffeoylquinic acid (chlorogenic acid), and cynaropicrin were obtained from Extrasynthese (Lyon, France), while cynarin (1,3-di-*O*-caffeoylquinic acid) was from Roth (Karlsruhe, Germany). Milli-Q system (Millipore Corp., Bedford, MA, USA) ultrapure water was used throughout the experimental study.

### 4.4. Statistical Analysis

Bartlett’s test was chosen to assess for homoscedasticity. Then, the data regarding morphological and bio-agronomical characterization were subjected to a two-way analysis of variance (ANOVA) based on a factorial combination of ‘GA_3_ treatment (2) × genotype (3)’. For the data on polyphenol profile characterization, a three-way ANOVA was adopted based on a factorial combination of ‘treatment (2) × genotype (3) × harvest time (2)’. Means were separated by the least significant difference (LSD) test when the *F*-test was significant. Statistical analysis was performed using the CoStat^®^ computer package version 6.003 (CoHort Software, Monterey, CA, USA).

## 5. Conclusions

Our results suggest that GA_3_ treatment improves the polyphenol profile of the globe artichoke, making it of interest as a primary source of a naturally occurring bioactive substance for fresh consumption or industrial (food or pharmaceutical) end-uses. In particular, it favored the accumulation of specific polyphenolic compounds in the waste material (i.e., apigenin derivatives in the floral stem, caffeoylquinic acids and cynaropicrin in the leaves), as well as in the edible fraction (particularly caffeoylquinic acids) when the heads were collected early. Indeed, our results highlight that the influence of GA3 treatment on the cynaropicrin and polyphenol profiles of globe artichoke plant parts is both harvest-time- and genotype-dependent. Among the studied genotypes, GA_3_ application was most effective in enhancing the total measured polyphenol and caffeoylquinic acid content in ‘Apollo’, regardless of the plant parts analyzed. In particular, the leaves of this genotype were the best accumulators of the main caffeoylquinic acids, i.e., 5-*O*- and 1,5-di-*O*-caffeoylquinic acids, as well as of luteolin derivatives, of which globe artichoke is one of the richest vegetable sources. GA_3_ treatment was also able to shorten the time to the first harvest (anticipating it by ~40 days in ‘Apollo’) for all the genotypes studied, while either maintaining (in ‘Apollo’ and ‘Violet de Provence’) or enhancing (in ‘Tema 2000’) the fresh weight yield. This is important for promoting local markets (with higher product prices) and managing export during the period from December to February. Thus, the significant occurrence of bioactive polyphenolic compounds in globe artichoke plant part, conjugated to the interesting productive traits obtained with GA_3_ supply, is important to satisfy the increased demand of functional foods by both fresh markets and industry. In particular, the stimulant effect of GA_3_ on secondary metabolite biosynthesis in globe artichoke has important implications for both its daily inclusion in the human diet and the utilization of waste material (floral stems and leaves) for natural antioxidant extraction to be used in the replacement of synthetic antioxidants. However, further studies are necessary to improve the knowledge regarding the effect of GA_3_ treatment on other genotypes of globe artichoke, possibly by verifying genotype-specific dose effects on nutraceutical and yield performances.

## Figures and Tables

**Table 1:** Mean square per each source of variation (percentage of total) resulting from analysis of variance for the content of total caffeoylquinic acid (Tot CQA), total apigenin (Tot Api), total luteolin (Tot Lut), total measured polyphenol (Tot Phe), and cynaropicrin (Cyn) in the floral stems (FSs), receptacles (REs) and leaves (LEs) of the globe artichokes.

Parameter	Source of Variation						
	Treatment (T)	Genotype (G)	Harvest Time (H)	T × G	T × H	G × H	T × G × H
FSs Tot CQA	24.9 ***	8.4 *	31.3 ***	13.2 ***	8.9 *	12.4 ***	0.9 ns
FSs Tot Api	18.6 ***	29.7 ***	18.4 ***	0.5 ns	20.8 ***	10.5 **	0.6 ns
FSs Tot Lut	17.2 ***	17.2 ***	22.2 ***	14.8 ***	1.7 ns	15.9 ***	1.0 ns
FSs Tot Phe	29.0 ***	25.9 ***	15.2 ***	4.9 *	14.4 ***	8.4 **	2.2 ns
REs Tot CQA	11.5 ***	43.6 ***	12.5 ***	13.7 ***	16.7 ***	0.5 ns	1.5 ns
REs Tot Api	1.9 ns	43.3 ***	25.1 ***	22.6 ***	3.0 ns	0.4 ns	3.5 ns
REs Tot Lut	16.1 ***	19.0 ***	32.0 ***	10.1 **	5.7 ns	15.2 ***	0.9 ns
REs Tot Phe	18.9 ***	33.5 ***	23.2 ***	0.8 ns	17.7 ***	2.8 ns	3.1 ns
LEs Tot CQA	0.5 ns	10.1 **	8.0 *	1.7 ns	18.6 **	59.1 ***	2.0 ns
LEs Tot Api	38.7 ***	37.1 ***	7.5 *	2.2 ns	5.6 ns	5.2 ns	3.7 ns
LEs Tot Lut	26.7 ***	24.4 ***	21.0 ***	0.2 ns	15.3 **	12.2 **	0.2 ns
LEs Tot Phe	16.5 ***	20.6 ***	11.3 **	12.0 **	26.7 ***	12.5 **	0.4 ns
LEs Cyn	12.7 **	9.9 **	31.4 ***	10.2 **	11.7 **	21.2 ***	2.9 ns
Df	1	2	1	2	1	2	2

***, **, and * indicate significance at *p* < 0.001, *p* < 0.01 and *p* < 0.05; ns: not significant. Df: degrees of freedom.

**Table 2:** Content (g kg^−1^ of D.M.) of total caffeoylquinic acid (Tot CQA), total apigenin (Tot Api), total luteolin (Tot Lut), total measured polyphenol (Tot Phe), and cynaropicrin (Cyn) in the floral stems (FSs), receptacles (REs), and leaves (LEs) of the globe artichokes as affected by the ‘GA_3_ treatment × harvest time’ interaction. Different letters within the same row show significant differences among means ± s.d. (LSD test, *p* < 0.05).

Parameter	H1		H2	
	GA_3_	Control	GA_3_	Control
FSs Tot CQA	14.65 ± 0.21 a	7.23 ± 0.26 b	5.89 ± 0.26 c	4.02 ± 0.15 d
FSs Tot Api	0.56 ± 0.06 b	0.31 ± 0.06 c	0.95 ± 0.09 a	0.59 ± 0.04 b
FSs Tot Lut	0.61 ± 0.04	0.24 ± 0.02	3.37 ± 0.16	5.25 ± 0.15
FSs Tot Phe	15.82 ± 0.22 a	7.79 ± 0.10 c	10.21 ± 0.28 b	9.86 ± 0.21 b
REs Tot CQA	2.10 ± 0.13 b	1.61 ± 0.16 c	1.67 ± 0.19 c	2.58 ± 0.12 a
REs Tot Api	1.32 ± 0.05	1.15 ± 0.09	1.11 ± 0.10	1.18 ± 0.09
REs Tot Lut	0.23 ± 0.05	0.19 ± 0.07	0.38 ± 0.06	0.49 ± 0.03
REs Tot Phe	3.65 ± 0.14 b	2.96 ± 0.19 c	3.17 ± 0.11 c	4.24 ± 0.13 a
LEs Tot CQA	1.42 ± 0.09 a	0.92 ± 0.10 c	0.86 ± 0.06 c	1.22 ± 0.16 b
LEs Tot Api	0.44 ± 0.02	0.90 ± 0.09	0.53 ± 0.06	0.89 ± 0.07
LEs Tot Lut	5.86 ± 0.20 a	2.29 ± 0.14 d	3.19 ± 0.16 c	4.36 ± 0.15 b
LEs Tot Phe	7.71 ± 0.16 a	4.11 ± 0.10 c	4.58 ± 0.15 c	6.47 ± 0.16 b
LEs Cyn	2.10 ± 0.11 b	1.70 ± 0.07 c	3.48 ± 0.15 a	2.61 ± 0.09 b

H1: harvest at winter; H2: harvest at early spring; GA_3_: gibberellic acid treatment; control: no gibberellic acid treatment.

**Table 3:** Content (g kg^−1^ of D.M.) of total caffeoylquinic acid (Tot CQA), total apigenin (Tot Api), total luteolin (Tot Lut), total polyphenol (Tot Phe), and cynaropicrin (Cyn) in the floral stems (FSs), receptacles (REs), and leaves (LEs) of the globe artichokes as affected by the ‘GA_3_ treatment × genotype’ interaction. Different letters within the same row show significant differences among means ± s.d. (LSD test, *p* < 0.05).

Parameter	GA_3_			Control		
	*Apollo*	*Tema 2000*	*Violet de Provence*	*Apollo*	*Tema 2000*	*Violet de Provence*
FSs Tot CQA	8.56 ± 0.59 c	11.03 ± 1.01 a	11.22 ± 1.02 a	3.17 ± 0.30d	3.97 ± 0.19 d	9.73 ± 1.00 b
FSs Tot Api	0.27 ± 0.02	1.41 ± 0.10	0.58 ± 0.05	0.24 ± 0.02	0.58 ± 0.03	0.53 ± 0.03
FSs Tot Lut	0.71 ± 0.05 d	3.23 ± 0.22 b	2.03 ± 0.15 c	0.28 ± 0.05 e	5.13 ± 0.48 a	2.83 ± 0.24 b
FSs Tot Phe	9.54 ± 0.68 c	15.67 ± 1.02 a	13.84 ± 1.19 b	3.69 ± 0.21 d	9.69 ± 0.28 c	13.09 ± 0.46 b
REs Tot CQA	1.20 ± 0.12 de	1.48 ± 0.09 cd	2.97 ± 0.23 b	0.99 ± 0.10 e	1.89 ± 0.11 c	3.40 ± 0.26 a
REs Tot Api	1.05 ± 0.05	1.05 ± 0.06	1.55 ± 0.08	0.65 ± 0.03	1.33 ± 0.10	1.52 ± 0.12
REs Tot Lut	0.49 ± 0.02	0.07 ± 0.01	0.36 ± 0.03	0.33 ± 0.03	0.32 ± 0.04	0.37 ± 0.03
REs Tot Phe	2.74 ± 0.21 d	2.60 ± 0.15 d	4.88 ± 0.41 b	1.98 ± 0.12 e	3.53 ± 0.21 c	5.29 ± 0.28 a
LEs Tot CQA	1.44 ± 0.10	1.10 ± 0.15	0.86 ± 0.05	1.39 ± 0.51	0.80 ± 0.06	1.02 ± 0.08
LEs Tot Api	0.44 ± 0.03	0.27 ± 0.02	0.74 ± 0.06	0.36 ± 0.02	1.62 ± 0.11	0.70 ± 0.04
LEs Tot Lut	5.67 ± 0.40 a	3.54 ± 0.30 c	4.36 ± 0.25 b	4.22 ± 0.32 b	2.53 ± 0.09 d	3.22 ± 0.04 c
LEs Tot Phe	7.56 ± 0.21 a	4.91 ± 0.28 c	5.96 ± 0.39 b	5.97 ± 0.28 b	4.95 ± 0.18 c	4.94 ± 0.31 c
LEs Cyn	2.47 ± 0.18 c	3.29 ± 0.15 a	2.60 ± 0.23 bc	1.80 ± 0.10 d	2.76 ± 0.05 b	1.91 ± 0.09 d

GA_3_: gibberellic acid treatment; control: no gibberellic acid treatment.

**Table 4:** Content (g kg^−1^ of D.M.) of total caffeoylquinic acid (Tot CQA), total apigenin (Tot Api), total luteolin (Tot Lut), total measured polyphenol (Tot Phe), and cynaropicrin (Cyn) in the floral stems (FSs), receptacles (REs) and leaves (LEs) as affected by the ‘harvest time × genotype’ interaction. Different letters within the same row show significant differences among means ± s.d. (LSD test, *p* < 0.05).

Parameter	H1			H2		
	*Apollo*	*Tema 2000*	*Violet de Provence*	*Apollo*	*Tema 2000*	*Violet de Provence*
FSs Tot CQA	5.59 ± 0.30 c	12.31 ± 1.06 b	14.93 ± 1.05 a	6.14 ± 0.38 c	2.70 ± 0.15 d	6.03 ± 0.19 c
FSs Tot Api	0.30 ± 0.02 c	0.85 ± 0.03 b	0.16 ± 0.01 d	0.22 ± 0.02 cd	1.14 ± 0.05 a	0.95 ± 0.05 ab
FSs Tot Lut	0.35 ± 0.04 c	0.50 ± 0.01 c	0.44 ± 0.04 c	0.64 ± 0.06 c	7.86 ± 0.29 a	4.43 ± 0.14 b
FSs Tot Phe	6.24 ± 0.15 d	13.66 ± 1.01 b	15.52 ± 0.39 a	6.99 ± 0.26 d	11.70 ± 1.05 c	11.41 ± 0.59 c
REs Tot CQA	1.04 ± 0.02 e	1.54 ± 0.04 d	2.98 ± 0.09 b	1.15 ± 0.09 e	1.83 ± 0.09 c	3.39 ± 0.22 a
REs Tot Api	0.47 ± 0.05	1.34 ± 0.08	1.90 ± 0.04	1.23 ± 0.13	1.04 ± 0.08	1.17 ± 0.09
REs Tot Lut	0.28 ± 0.01 b	0.12 ± 0.01 c	0.24 ± 0.03 b	0.54 ± 0.06 a	0.27 ± 0.01 b	0.49 ± 0.01 a
REs Tot Phe	1.79 ± 0.09	2.99 ± 0.14	5.12 ± 0.41	2.92 ± 0.13	3.14 ± 0.17	5.05 ± 0.23
LEs Tot CQA	0.73 ± 0.05 c	1.30 ± 0.05 b	1.47 ± 0.09 b	2.10 ± 0.09 a	0.60 ± 0.03 cd	0.42 ± 0.01 d
LEs Tot Api	0.38 ± 0.01	1.12 ± 0.09	0.51 ± 0.05	0.43 ± 0.03	0.77 ± 0.03	0.92 ± 0.05
LEs Tot Lut	4.66 ± 0.35 bc	3.47 ± 0.05 d	4.08 ± 0.25 c	5.23 ± 0.22 a	2.60 ± 0.21 e	3.50 ± 0.32 d
LEs Tot Phe	5.77 ± 0.30 c	5.89 ± 0.21 c	6.07 ± 0.19 bc	7.76 ± 0.52 a	3.97 ± 0.35 e	4.84 ± 0.20 d
LEs Cyn	1.99 ± 0.12 c	1.46 ± 0.05 d	2.24 ± 0.09 b	2.27 ± 0.20 b	4.59 ± 0.32 a	2.26 ± 0.12 b

H1: harvest at winter; H2: harvest at early spring.

**Table 5:** Polyphenol profiles (g kg^−1^ of D.M.) of the floral stems (FSs), receptacles (REs) and leaves (LEs) of globe artichokes as affected by the main effects under study. Different letters within the same row and factor under study show significant differences among means ± s.d. (LSD test, *p* < 0.05) (when s.d. < 0.001, it is no reported).

Compound	Treatment		Genotype			Harvest Time	
	GA_3_	Control	*Apollo*	*Tema 2000*	*Violet de Provence*	H1	H2
FSs 5-CQA	4.76 ± 0.32 a	3.12 ± 0.11 b	2.03 ± 0.12 c	3.97 ± 0.22 b	5.81 ± 0.38 a	5.20 ± 0.21 a	2.68 ± 0.20 b
FSs 3,5-diCQA	2.24 ± 0.15 a	1.01 ± 0.08 b	1.30 ± 0.11 b	1.74 ± 0.14 a	1.84 ± 0.12 a	2.36 ± 0.14 a	0.90 ± 0.08 b
FSs 1,5-diCQA	3.18 ± 0.25 a	1.44 ± 0.08 b	2.39 ± 0.21 b	1.83 ± 0.09 c	2.70 ± 0.20 a	3.32 ± 0.09 a	1.30 ± 0.05 b
FSs 4,5-diCQA	0.11 a	0.07 b	0.14	nd	0.13	0.07	0.11
FSs API-7-rut	nd	0.01	0.01	tr	tr	0.01	tr
FSs API-7-glc	0.06 a	0.02 b	0.11	nd	tr	0.07	nd
FSs API-7-mal	0.69 ± 0.05 a	0.41 ± 0.03 b	0.10 c	1.00 ± 0.08 a	0.56 ± 0.03 b	0.34 b	0.76 ± 0.03 a
FSs API	0.07 a	0.02 b	0.13	tr	tr	0.02 b	0.06 a
FSs LUT-7-glc	1.06 ± 0.06 b	1.66 ± 0.11 a	0.16 c	2.52 ± 0.23 a	1.40 ± 0.11 b	0.23 b	2.49 ± 0.16 a
FSs LUT-7-glr	0.75 ± 0.02 b	1.04 ± 0.09 a	0.03 c	1.66 ± 0.15 a	1.00 ± 0.05 b	0.12 b	1.67 ± 0.12 a
FSs LUT-7-mal	0.08 a	0.04 b	0.14	nd	0.04	0.03 b	0.09 a
FSs LUT	0.05	tr	0.08	tr	nd	0.05	nd
REs 5-CQA	0.62 ± 0.05	0.61 ± 0.05	0.33 ± 0.01 b	0.28 b	1.24 ± 0.11 a	0.56 ± 0.04 b	0.67 a
REs 3,5-diCQA	0.42 ± 0.01 b	0.64 ± 0.03 a	0.35 ± 0.02 b	0.60 a	0.65 ± 0.03 a	0.47 b	0.60 ± 0.04 a
REs 1,5-diCQA	0.63 ± 0.01 b	0.95 ± 0.03 a	0.39 ± 0.01 c	0.68 ± 0.04 b	1.30 ± 0.09 a	0.72 ± 0.03 b	0.86 ± 0.03 a
REs API-7-rut	0.32 ± 0.03 b	0.63 ± 0.05 a	0.32 c	0.48 b	0.63 ± 0.02 a	0.60 a	0.35 b
REs API-7-glc	0.43 ± 0.02 a	0.25 b	0.23 b	0.14 b	0.65 ± 0.01 a	0.38 a	0.29 ± 0.02 b
REs API-7-glr	tr	0.12	tr	0.08	0.10	0.08 a	0.03 b
REs API	0.27 ± 0.01	0.20	0.31 a	0.34 ± 0.02 a	0.06 b	0.18 b	0.29 a
REs LUT-7-glc	0.25 ± 0.02	0.23 ± 0.02	0.28 ± 0.01 a	0.12 b	0.33 ± 0.01 a	0.16 b	0.32 ± 0.02 a
REs LUT-7-mal	0.07	0.07	0.14 a	0.04 b	0.03 b	0.05 b	0.09 a
LEs 5-CQA	0.68 ± 0.01 b	0.85 ± 0.02 a	0.90 ± 0.02 a	0.70 ± 0.05 b	0.70 b	0.89 ± 0.02 a	0.64 ± 0.01 b
LEs 3,5-diCQA	0.12	0.11	0.13	0.12	0.10	0.11	0.13
LEs 1,5-diCQA	0.16 ± 0.01 b	0.28 ± 0.01 a	0.39 ± 0.01 a	0.13 b	0.14 b	0.17 b	0.27 a
LEs API-7-rut	0.35 ± 0.02	0.31	0.21 b	0.29 b	0.48 a	0.33 ± 0.01	0.33 ± 0.02
LEs API-7-glc	0.27 ± 0.01	0.25	0.19 b	0.44 a	0.15 b	0.22 ± 0.02 b	0.30 a
LEs API-7-glr	0.16 ± 0.01 a	0.05 b	tr	0.22 ± 0.01 a	0.09 b	0.13 a	0.08 b
LEs LUT-7-glc	2.25 ± 0.15	2.05 ± 0.13	2.63 ± 0.09 a	2.20 ± 0.11 b	1.62 ± 0.13 c	2.20 ± 0.17	2.10 ± 0.16
LEs LUT-7-glr	0.67 ± 0.02 a	0.52 ± 0.03 b	0.53 ± 0.03 b	0.33 ± 0.04 c	0.92 ± 0.08 a	0.56 ± 0.04	0.63 ± 0.01
LEs LUT-7-mal	1.01 ± 0.05	0.90 ± 0.02	1.10 ± 0.08 a	0.52 ± 0.01 b	1.25 ± 0.09 a	1.31 ± 0.13 a	0.61 ± 0.05 b
LEs LUT	tr	0.46 ± 0.01	0.69 ± 0.03	tr	tr	tr	0.46 ± 0.03
LEs Cyn	2.84 ± 0.09 a	2.11 ± 0.10 b	2.13 ± 0.15 b	3.02 ± 0.23 a	2.26 ± 0.10 b	1.90 ± 0.06 b	3.04 ± 0.16 a

tr: trace; nd: not detected. H1: harvest at winter; H2: harvest at early spring; GA_3_: gibberellic acid treatment; control: no gibberellic acid treatment. DM: dry matter; 5-CQA: 5-*O*-caffeoylquinic acid (or chlorogenic acid); 3,5-diCQA: 3,5-di-*O*-caffeoylquinic acid; 1,5-diCQA: 1,5-di-*O*-caffeoylquinic acid; 4,5-diCQA: 4,5-di-*O*-caffeoylquinic acid; API-7-rut: Apingenin 7-*O*-rutinoside; API-7-glc: Apigenin 7-*O*-glucoside; API-7-glr: Apigenin 7-*O*-glucuronide; API-7-mal: Apigenin 7-*O*-malonylglucoside; API: Apigenin; LUT-7-glc: Luteolin 7-*O*-glucoside; LUT-7-glr: Luteolin 7-*O*-glucuronide; LUT-7-mal: Luteolin 7-*O*-malonylglucoside; LUT: Luteolin; Cyn: cynaropicrin.

**Table 6:** Mean square per each source of variation (percentage of total) resulting from the analysis of variance on the parameters relating to earliness, yield response, and head bio-morphological characterization of the globe artichoke. ***, **, * indicate significance at *p* < 0.001, *p* < 0.01 and *p* < 0.05.

Parameter	Source of Variation		
	Treatment (T)	Genotype (G)	T × G
Earliness	14.0 ***	79.2 ***	6.8 ***
Number of heads plant^−1^	66.0 ***	10.9 ***	23.1 ***
Fresh weight yield	12.2 *	72.9 ***	14.9 *
Mean head fresh weight	6.2 *	64.4 ***	29.4 **
Length/width head ratio	11.1 *	61.2 ***	27.8 **
Edible fraction percentage	17.6 **	42.5 **	39.9 ***
**Degrees of freedom**	1	2	2

**Table 7:** Plant growth response and head bio-morphological characterization of globe artichokes as affected by the ‘GA_3_ treatment × genotype’ interaction. Different letters within the same parameter show significant differences among means ± s.d. (LSD test, *p* < 0.05).

Genotype	Earliness (d) *		Number of Heads Plant^−1^		Fresh Weight Yield (Kg Plant^−1^)	
	Control	GA_3_	Control	GA_3_	Control	GA_3_
*Apollo*	234 ± 5 a	194 ± 4 b	12.4 ± 1.0 b	13.3 ± 0.6 a	3.3 ± 0.1 a	3.5 ± 0.3 a
*Tema 2000*	157 ± 6 c	130 ± 5 f	8.1 ± 0.5 d	13.9 ± 0.5 a	1.1 ± 0.1 c	1.6 ± 0.1 b
*Violet de Provence*	144 ± 8 de	136 ± 4 ef	11.6 ± 0.7 c	12.9 ± 0.4 b	1.7 ± 0.2 b	1.8 ± 0.2 b
	**Mean Head Fresh Weight (g)**		**Length/Width Head Ratio**		**Edible Fraction Percentage (%)**	
	**Control**	**GA_3_**	**Control**	**GA_3_**	**Control**	**GA_3_**
*Apollo*	269 ± 5 a	261 ± 5 a	0.99 ± 0.02 e	1.14 ± 0.04 d	20.7 ± 1.1 c	25.0 ± 1.3 a
*Tema 2000*	141 ± 4 cd	119 ± 6 e	1.40 ± 0.04 b	1.47 ± 0.06 a	19.7 ± 0.8 d	20.0 ± 1.1 cd
*Violet de Provence*	155 ± 6 bc	132 ± 3 d	1.32 ± 0.05 c	1.40 ± 0.07 b	22.0 ± 0.9 b	21.7 ± 1.2 b

* Expressed as the number of days between transplanting and the harvest of the main head.

## Data Availability

All data are available via email request to the corresponding author.
